# Computed tomography-based conventional imaging features and texture analysis characteristics of chemotherapy drug-related acute pancreatitis

**DOI:** 10.3389/fmed.2025.1497944

**Published:** 2025-05-12

**Authors:** Quanlin Li, Chuanmei Liu, Zijian Fan, Hong Zhang, Zhiguo Kai

**Affiliations:** ^1^Department of Radiology, Shandong University of Traditional Chinese Medicine Affiliated Hospital, Jinan, China; ^2^Department of Radiology, The 4th People’s Hospital of Jinan, Jinan, China

**Keywords:** chemotherapy, cancer, acute pancreatitis, computed tomography, imaging

## Abstract

**Purpose:**

Chemotherapy drug-related acute pancreatitis (CDRAP) is a rare adverse event that poses significant challenges to clinicians. This study aimed to describe plain computed tomography (CT)- and contrast-enhanced computed tomography (CECT)-based conventional imaging features and texture analysis characteristics of CDRAP.

**Methods:**

A total of 62 patients with initial clinical and/or biochemical evidence of pancreatitis and 34 patients with normal pancreatic manifestations who underwent CT during chemotherapy were retrospectively included. The diagnosis of CDRAP was established based on clinical, imaging, and biochemical findings. Conventional imaging features, texture analysis characteristics, clinical and biochemical parameters, other complications, chemotherapy drugs, and patient outcomes related to CDRAP were recorded.

**Results:**

A total of 20 (32.26%) patients who were clinically diagnosed with CDRAP had normal pancreatic morphology on CT, while 42 (67.74%) patients presented with changes indicative of acute pancreatitis. The CT findings of 62 CDRAP cases were as follows: diffuse (*n* = 19) or focal (*n* = 21) pancreatic enlargement, diffuse (*n* = 12) or focal (*n* = 4) heterogeneous enhancement, peripancreatic stranding (*n* = 20), acute peripancreatic fluid collection (*n* = 10), and pseudocyst (*n* = 2). A total of 17 texture features were identified to differentiate CDRAP from normal pancreatic manifestations.

**Conclusion:**

CDRAP mainly manifested as interstitial edematous pancreatitis with/without normal pancreatic morphology on CT. Imaging texture analysis may serve as a potential biomarker for its detection. By combining conventional imaging features with texture analysis characteristics, there is potential to assist radiologists and clinicians in the identification of CDRAP, thereby improving the quality of life for cancer patients.

## Introduction

The incidence of cancer has been increasing rapidly; however, the overall cancer mortality rate is declining, mainly due to significant advances in cancer therapy (1–3). Chemotherapy and the use of combinations of antitumor agents remain the mainstay of treatment for the majority of solid and hematological malignancies worldwide, including acute lymphocytic leukemia (ALL), lymphoma, and non-small-cell lung cancer (4–6). Combination chemotherapy can sensitize cancer cells to drugs, modulate signaling pathways, and combat multidrug resistance (7). However, chemotherapy-related adverse events (AEs) are common in clinical practice, as defined by the United States National Cancer Institute’s (NCI) Common Terminology Criteria for AEs (8). As some AEs can significantly impact patient management and prognosis, it is essential for radiologists to recognize the radiological appearances of chemotherapy drug-related AEs. Imaging features of many frequently encountered chemotherapy-related AEs affecting multiple organs have been described, including hepatitis (9), thyroid dysfunction (10), and cardiac ischemia (11).

Chemotherapy drug-related acute pancreatitis (CDRAP) is a rare adverse event that often poses significant challenges for clinicians (12). Computed tomography (CT) is considered the best imaging modality for pancreatitis in terms of performance and reproducibility (13). Limited studies, primarily consisting of a few case reports (14–17), have described the imaging features of drug-related pancreatitis as diagnostic evidence. For example, Yang et al. (17) reported a case of acute pancreatitis induced by combination chemotherapy used in the treatment of acute myeloid leukemia, which presented with a swollen pancreas with blurred edges and thickened left prerenal fascia. M’harzi et al. (18) described a case of CDRAP characterized by pancreatic swelling, loss of physiological lobulation, and significant infiltration of the surrounding fat. Nevertheless, the systematic radiological characterization of CDRAP remains poorly understood. In addition, radiomics is increasingly used to extract multi-texture features from medical images, and by analyzing the distribution and relationship of pixel or voxel greyscales in images, it is possible to objectively evaluate the features of tissues that are indistinguishable to the naked eye (19). The texture analysis characteristics of CDRAP have not been elucidated. Early detection of abnormal CT features in patients with CDRAP could facilitate the timely adjustment of treatment plans and improve patient prognosis.

Therefore, the objective of this study is to explore the CT-based conventional imaging features and texture analysis characteristics of CDRAP and to provide evidence for clinicians.

## Methods

### Patients

**Figure 1:**
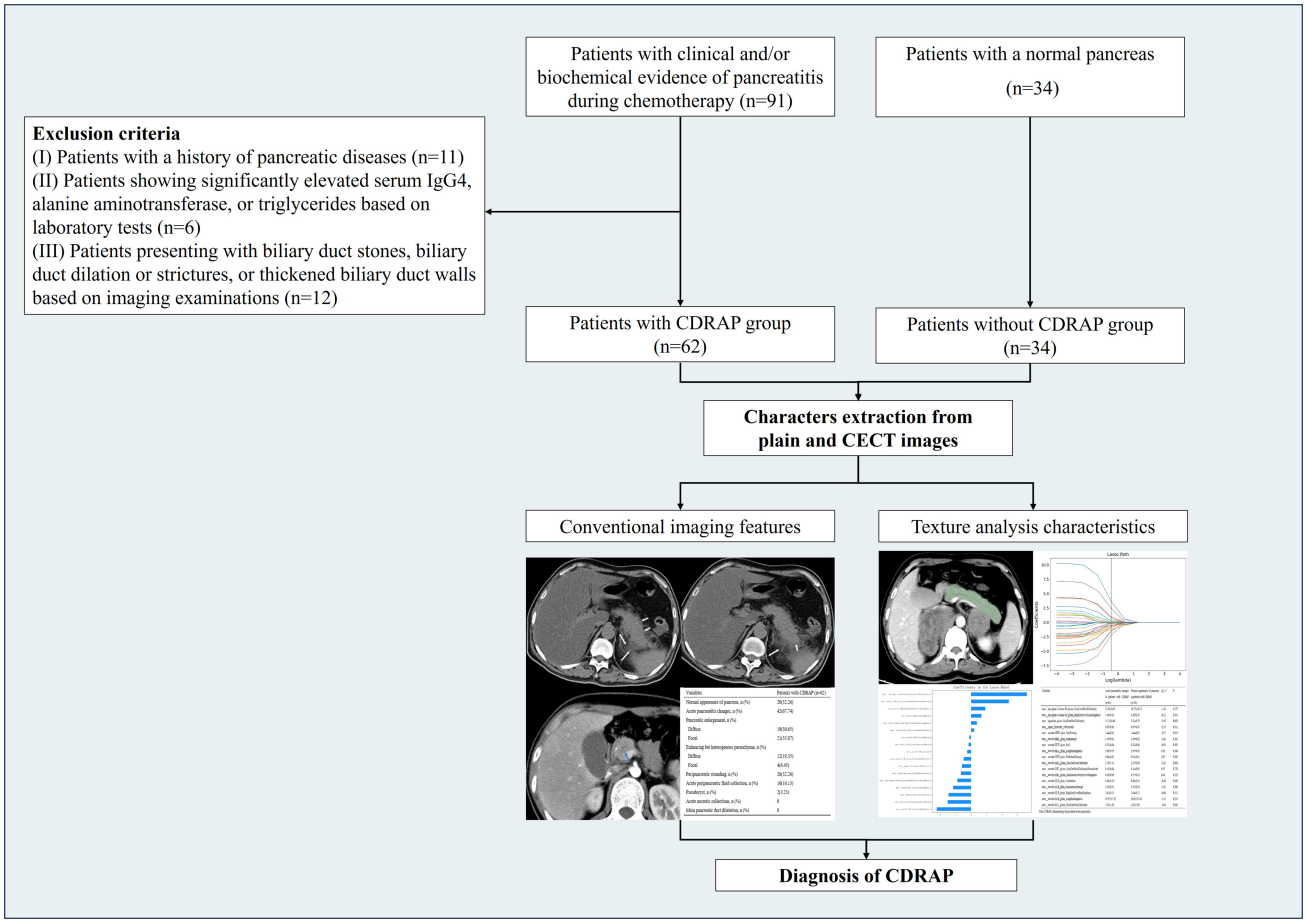
Flowchart.

This retrospective study was approved by the ethics committee (QL2021-230). Given the retrospective design of the study and the use of anonymized patient data, the requirement for informed consent was waived. Patients with clinical and/or biochemical evidence of pancreatitis during chemotherapy (CDRAP group) and patients with a normal pancreas (non-CDRAP group) were retrospectively included, and all underwent plain CT and contrast-enhanced CT (CECT). The inclusion criteria for the CDRAP group were as follows: (I) a clinical diagnosis of acute pancreatitis, meeting at least two of the following criteria (20): (a) typical abdominal pain, (b) serum amylase (AMY) and/or lipase (LIP) levels elevated to more than three times the upper limit of normal, and (c) imaging findings consistent with acute pancreatitis; (II) acute pancreatitis occurring for the first time during chemotherapy treatment for malignant tumors. The exclusion criteria were as follows: (I) Patients with a history of pancreatic diseases; (II) patients with significantly elevated levels of serum Immunoglobulin G4 (IgG4), alanine aminotransferase, or triglycerides, as indicated by laboratory tests; and (III) patients presenting with biliary duct stones, biliary duct dilation or strictures, or thickened biliary duct walls, as observed in imaging examinations. Based on these criteria, 92 patients with CDRAP were eligible for inclusion; however, 62 (36 male and 26 female participants) were ultimately enrolled in this cross-sectional study after screening (Figure 1).

Demographic information (hypertension, history of alcohol consumption, and history of smoking), comorbidities (history of cholelithiasis and hypertriglyceridemia), biochemical parameters (AMY, LIP, total cholesterol, triglycerides, low-density lipoprotein cholesterol [LDL-C], high-density lipoprotein cholesterol [HDL-C], alanine aminotransferase, aspartate aminotransferase, bilirubin, creatinine, hemoglobin, neutrophil count, lymphocyte count, and platelet count), primary cancer type, and the number of chemotherapy cycles were obtained from the hospital information database. The neutrophil-to-lymphocyte ratio (NLR) was calculated as neutrophil count/ lymphocyte count. The platelet-to-lymphocyte ratio (PLR) was calculated as platelet count/ lymphocyte count. The systemic immune-inflammation index (SII) was calculated as platelet count× neutrophil count/ lymphocyte count. Other chemotherapy-related AEs, including hematologic toxicity, gastrointestinal toxicity, nephrotoxicity, and neurologic toxicity, were recorded according to the Common Terminology Criteria for AEs (version 5.0) (21).

For patients with CDRAP, clinical symptomatology (abdominal pain, nausea, vomiting, and diarrhea), the time from the initiation of chemotherapy to the onset of the clinical symptoms of acute pancreatitis, and the time from the onset of the clinical symptoms to the CT examination were also recorded.

### Severity classification of acute pancreatitis

The classification of the severity of acute pancreatitis is as follows: (a) mild acute pancreatitis: this is characterized by the absence of organ failure and no local or systemic complications; (b) moderately severe acute pancreatitis: this is characterized by the presence of organ failure that resolves within 48 h (transient organ failure) and/or local or systemic complications, but without persistent organ failure; and (c) severe acute pancreatitis: this is characterized by persistent organ failure that lasts more than 48 h and may involve single or multiple organs (13).

### Drug classification categories

Commonly used drugs for various diseases have been reported to cause acute pancreatitis. Drug classification categories were based on the classification criteria proposed by Badalov et al. (22), which summarize the associated drugs as follows: (a) Class Ia included drugs with at least one case report, evidence of a positive rechallenge, and exclusion of other causes of acute pancreatitis such as cholelithiasis, alcohol, and hypertriglyceridemia; (b) Class Ib included drugs with at least one case report and evidence of a positive rechallenge but without exclusion of other causes of acute pancreatitis; (c) Class II included drugs with at least four case reports, where the latency period was consistent in at least 75% of cases; (d) Class III included drugs with at least two case reports but without rechallenge data or a consistent latency period; and (e) Class IV included drugs with only one case report and no rechallenge data.

### CT imaging

Plain and CECT images (256-Sclice Brilliance iCT, Philips Medical Systems, Amsterdam, The Netherlands) were obtained before and after injecting intravenous (IV) contrast iopromide (300 mg/I/mL, Bayer HealthCare, Germany) at a flow rate of 3.0 mL/s. Cross-sectional imaging was performed during the portal venous phase at 60–80 s, with imaging parameters set at 120 kV, 200 mAs, and a slice thickness of 5.0 mm.

### Imaging review and texture analysis

The Atlanta criteria (13) were used to evaluate CT and CECT images of the patients with CDRAP. The conventional imaging features assessed included the following: (a) focal or diffuse pancreatic enlargement, (b) focal or diffuse enhancing but heterogeneous parenchyma, (c) peripancreatic stranding, (d) acute peripancreatic fluid collection, (e) pseudocyst, (f) acute necrotic collections, and (g) main pancreatic duct dilatation. Double-blind independent assessments were performed by two radiologists, one with 5 years of experience in diagnosing abdominal diseases (author 1) and the other with 6 years of experience (author 2). A third radiologist with 30 years of experience reviewed the images when the two radiologists could not reach an agreement, and a consensus was subsequently obtained.

The two radiologists used open-source 3D Slicer software, version 4.13.1,^1^ to perform texture analysis on 5-mm axial images of the venous portal phase. The maximum axial level of the pancreas was selected, and the region of interest (ROI) was manually drawn along the edges of the pancreas (Supplementary Figure 1). All texture parameters (first-, second- and higher-order statistics) were extracted and analyzed.

### Statistical analysis

Statistical analysis was performed using SPSS version 22.0 (IBM, Armonk, NY, USA). Continuous variables were expressed as mean ± standard deviation, and categorical variables were expressed as counts with proportions (%). Intergroup comparisons of continuous variables were performed using the independent samples *t*-test or the Mann–Whitney U test, while the categorical variables were analyzed using the chi-squared test. LASSO regression was employed to identify the texture analysis features that differentiated CDRAP from normal pancreatic manifestations. Logistic regression analysis was conducted to evaluate the risk factors associated with chemotherapy-related AEs. Intraclass correlation coefficients (ICCs) were used to assess interobserver variability in the texture analysis. ICC values<0.40 signified poor agreement; values between 0.41 and 0.60 indicated moderate agreement; values from 0.61 to 0.80 represented good agreement; and values >0.80 reflected excellent agreement. Differences with a *p*-value of <0.05 were considered statistically significant.

## Results

### Clinical features

A total of 62 patients (36 male and 26 female participants; median age 51.19 ± 19.53 years) with CDRAP and 34 patients with normal pancreatic manifestations (16 male and 28 female participants; median age 53.70 ± 15.70 years) were included in this study (Table 1). All patients in the CDRAP group were diagnosed with primary tumors, including lymphoma (*n* = 30), stomach cancer (*n* = 8), lung cancer (*n* = 8), ALL (*n* = 4), breast cancer (*n* = 2), esophageal cancer (*n* = 4), multiple myeloma (*n* = 2), ovarian cancer (*n* = 2), and thyroid cancer (*n* = 2). In addition, lymphoma (*n* = 18), stomach cancer (*n* = 5), lung cancer (*n* = 4), ALL (*n* = 4), breast cancer (*n* = 1), and esophageal cancer (*n* = 2) were identified in patients without CDRAP. The average levels of AMY and LIP in the CDRAP group were 435.40 ± 415.02 U/L (normal 30–110 U/L) and 838.45 ± 683.39 U/L (normal 23–300 U/L), respectively. Patients with clinical symptoms of pancreatitis experienced stomachache (*n* = 54), nausea (*n* = 16), vomiting (*n* = 10), and diarrhea (*n* = 4).

**Table 1:** Baseline demographics of the patients with and without CDRAP.

Variables	Patients with CDRAP (*n* = 62)	Patients without CDRAP (*n* = 34)	*t*/*Z*/*χ*^2^	*p*-value
Age, years	51.19 ± 19.53	53.70 ± 15.70	−0.14	0.890
Sex, *n* (%)			1.07	0.301
Male	36 (58.06)	16 (47.06)		
Female	26 (41.94)	18 (52.94)		
Hypertension, *n* (%)	20 (32.26)	6 (17.65)	2.37	0.123
History of alcohol consumption, *n* (%)	12 (19.35)	4 (11.76)	0.911	0.340
History of smoking, *n* (%)	20 (32.26)	10 (29.41)	0.083	0.774
History of cholelithiasis, *n* (%)	4 (6.45)	0	2.289	0.130
Hypertriglyceridemia, *n* (%)	18 (29.03)	8 (23.53)	0.337	0.562
Primary cancer, *n* (%)			4.22	0.837
Lymphoma	30 (48.39)	18 (52.94)		
Stomach cancer	8 (12.90)	5 (14.71)		
Lung cancer	8 (12.90)	4 (11.76)		
Acute lymphocytic leukemia	4 (6.45)	4 (11.76)		
Esophageal cancer	4 (6.45)	2 (5.88)		
Multiple myeloma	2 (3.23)	0		
Breast cancer	2 (3.23)	1 (2.94)		
Ovarian cancer	2 (3.23)	0		
Thyroid cancer	2 (3.23)	0		
Number of chemotherapy cycle	3.61 ± 2.98	3.41 ± 1.74	−0.65	0.514
Clinical symptoms, *n* (%)				NA
Stomachache	54 (87.10)	NA		
Nausea	16 (25.81)	NA		
Vomiting	10 (16.13)	NA		
Diarrhea	4 (6.45)	NA		
Serum AMY, U/L	435.40 ± 415.02	NA		
Serum LIP U/L	838.45 ± 683.39	NA		
Total cholesterol, mmol/L	4.08 ± 1.30	4.29 ± 1.13	−0.42	0.678
Triglyceride, mmol/L	1.36 ± 0.72	1.49 ± 0.49	−1.54	0.122
LDL-C, mmol/L	2.53 ± 1.58	2.64 ± 0.93	−1.54	0.122
HDL-C, mmol/L	1.26 ± 0.95	0.97 ± 0.30	−1.02	0.309
Alanine Aminotransferase, U/L	60.23 ± 78.31	26.44 ± 31.27	−2.35	0.019
Aspartate Aminotransferase, U/L	54.70 ± 56.50	33.87 ± 31.27	−2.24	0.025
Bilirubin, μmol/L	28.96 ± 40.29	18.05 ± 25.82	−1.30	0.195
Creatinine, μmol/L	90.03 ± 126.99	59.25 ± 14.03	−0.07	0.948
Hemoglobin, g/L	107.94 ± 16.03	119.82 ± 21.94	−3.01	0.003
NLR	5.71 ± 7.58	5.47 ± 6.77	−0.47	0.641
PLR	236.69 ± 150.49	279.75 ± 289.07	−0.24	0.808
SII	1159.76 ± 902.77	1803.62 ± 2691.20	−0.37	0.714
Chemotherapy-related AEs, *n* (%)	30 (48.39)	18 (52.94)	0.18	0.670
Hematologic toxicity	26 (41.94)	10 (29.41)	1.47	0.225
Gastrointestinal toxicity	16 (25.81)	10 (29.41)	0.15	0.704
Nephrotoxicity	6 (9.68)	0	3.51	0.061
Neurologic toxicity	2 (3.23)	2 (5.88)	0.39	0.533

CDRAP, chemotherapy drug-related acute pancreatitis; AMY, serum amylase; LIP, serum lipase; LDL-C, low-density lipoprotein cholesterol; HDL-C, high-density lipoprotein cholesterol; NLR, neutrophil to lymphocyte ratio; PLR, platelet to lymphocyte ratio; SII, systemic immune inflammation index; AE, adverse event.

The comparison of demographic characteristics, comorbidities, and biochemical indicators between the patients with and without CDRAP is shown in Table 1. There was a significant difference in alanine aminotransferase, aspartate aminotransferase, and hemoglobin levels between the two groups (*p* < 0.05). In addition, 30 out of 62 (48.39%) patients with CDRAP experienced other chemotherapy-related AEs, including hematologic toxicity (*n* = 26), gastrointestinal toxicity (*n* = 16), nephrotoxicity (*n* = 6), and neurologic toxicity (*n* = 2). Furthermore, 18 out of 34 (52.94%) patients without CDRAP experienced other chemotherapy-related AEs, including hematologic toxicity (*n* = 10), gastrointestinal toxicity (*n* = 10), and neurologic toxicity (*n* = 2).

### Conventional CT imaging and texture analysis features

The conventional CT imaging features of CDRAP are summarized in Table 2. Of the 62 clinically diagnosed CDRAP patients, 20 (32.26%) showed a normal pancreas on CT, while 42 (67.74%) presented with manifestations of acute pancreatitis. Pancreatic enlargement (Supplementary Figures 2, 3) was seen in 40 of the 62 (64.52%) patients and was the most common CT feature of acute pancreatitis (diffuse = 19 and focal = 21). Heterogenous enhancement was identified in 16 of the 62 (25.81%) patients (diffuse = 12 and focal = 4). Regarding the other CT features, 20 (32.26%) patients exhibited peripancreatic strands (Supplementary Figure 2), 10 (16.13%) patients had acute peripancreatic fluid collections (Supplementary Figure 3), and two (3.23%) patients had pseudocysts. No acute necrotic collection or main pancreatic duct dilatation was found in these patients.

**Table 2:** Conventional CT findings of CDRAP after treatment with chemotherapy.

Variables	Patients with CDRAP (*n* = 62)
Normal appearance of the pancreas, *n* (%)	20 (32.26)
Acute pancreatitis changes, *n* (%)	42 (67.74)
Pancreatic enlargement, *n* (%)	
Diffuse	19 (30.65)
Focal	21 (33.87)
Enhancing but heterogeneous parenchyma, *n* (%)	
Diffuse	12 (19.35)
Focal	4 (6.45)
Peripancreatic stranding, *n* (%)	20 (32.26)
Acute peripancreatic fluid collection, *n* (%)	10 (16.13)
Pseudocyst, *n* (%)	2 (3.23)
Acute necrotic collections, *n* (%)	0
Main pancreatic duct dilatation, *n* (%)	0

The imaging features were assessed as per the Atlanta criteria. Acute necrotic collections include pancreatic necrosis and peripancreatic necrosis.

A total of 17 texture features were extracted and analyzed for each patient, including 1 first-order, 5 gray-level co-occurrence matrix (GLCM), 3 gray-level run length matrix (GLRLM), 7 gray-level size zone matrix (GLSZM), and 1 gray-level dependence matrix (GLDM) parameters (Supplementary Figure 4, Supplementary Table 1). Inter-observer variability analysis between the two radiologists showed good to excellent agreement (ICC > 0.6) for all texture parameters.

Given that 32.26% of clinically diagnosed CDRAP patients demonstrated normal pancreatic morphology on CT imaging and that 67.74% exhibited radiological manifestations of acute pancreatitis, a comparative analysis of texture analysis features was conducted between these subgroups. The analysis revealed statistically significant differences in three GLSZM features (auto__logarithm_glszm_SizeZoneNonUniformity, auto__wavelet-LLL_glszm_SizeZoneNonUniformity, and auto__wavelet-LHL_glszm_SizeZoneNonUniformity), one first-order statistical feature (auto__square_firstorder_10Percentile), and one GLCM feature (auto__wavelet-HHH_glcm_SumEntropy) between the two cohorts (Supplementary Table 2).

### Management and outcomes

In terms of management and outcomes (Table 3), the mean time interval from the initiation of chemotherapy to the development of clinical symptoms of acute pancreatitis was 181.33 ± 179.70 days. The mean time interval from clinically suspected acute pancreatitis to CT examination was 66.33 ± 60.72 h.

**Table 3:** Management and outcomes of patients with CDRAP.

Follow-up of patients with acute pancreatitis	Patients with CDRAP (*n* = 62)
Time from the initiation of chemotherapy to the onset of the clinical symptoms of acute pancreatitis, days	181.33 ± 179.70
Time from the onset of clinically suspected acute pancreatitis to CT examination, hours	66.33 ± 60.72
Following the resolution of pancreatic development, *n* (%)	
Pseudocyst	10 (16.13)
Pancreatic atrophy	21 (33.87)
Pancreatic exocrine dysfunction (diarrhea or steatorrhea)	4 (6.45)
Pancreatic endocrine dysfunction	0

CDRAP, chemotherapy drug-related acute pancreatitis.

**Figure 2:**
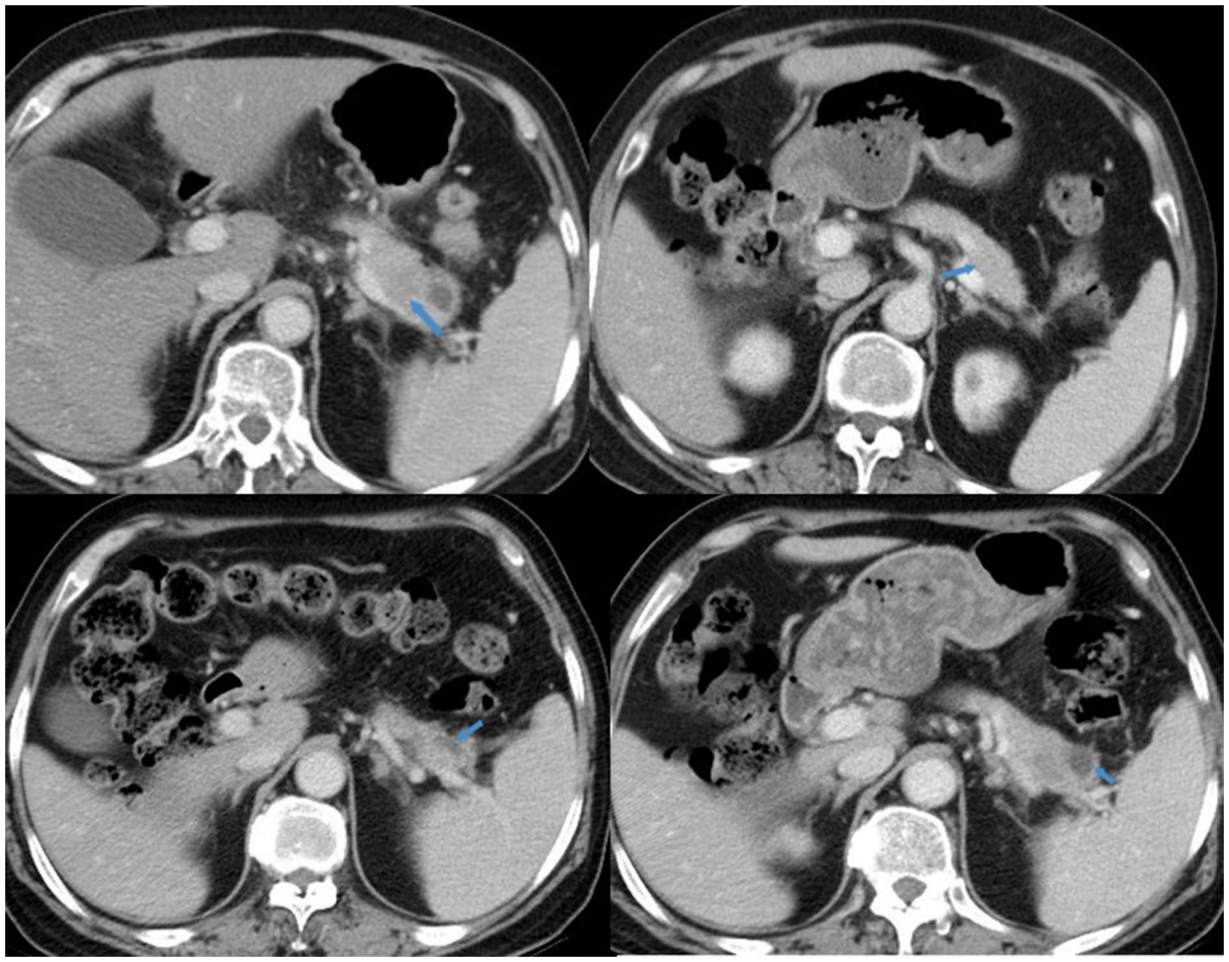
A 65-year-old woman with lung cancer. She received a docetaxel and cisplatin regimen for two cycles, with AMY = 449 U/L. CT imaging showed pancreatic changes, from heterogeneous enhancement (2019-5-31, upper right) to lesion area reduction and improvement (2019-12-31, upper left), followed by eventual pseudocyst formation (2020-3-19 and 2020-6-30, lower right and left).

Follow-up imaging features were recorded for 62 CDRAP patients. A total of 21 (33.87%) patients developed pancreatic atrophy, 4 patients developed pancreatic exocrine dysfunction, 2 patients presented with diarrhea, and 2 patients presented with both diarrhea and steatorrhea. None of these patients developed pancreatic endocrine dysfunction, such as new-onset diabetes mellitus. In addition, of the 20 patients who initially had normal pancreases, 4 developed acute pancreatitis—two presented with focal pancreas enlargement and the other two presented with heterogeneous enhancement (Supplementary Figure 5). The remaining 16 patients consistently showed a morphologically normal pancreas on follow-up imaging. Furthermore, one female patient presented with the progression of pancreatic changes, from heterogeneous enhancement to improvement, followed by eventual pseudocyst formation approximately 1 year after the first diagnosis of acute pancreatitis, as shown in Figure 2.

At a mean follow-up period of 75.60 ± 42.85 days, the biochemical parameters of CDRAP patients were reassessed. The results demonstrated a statistically significant difference in levels of AMY, total cholesterol, alanine aminotransferase, aspartate aminotransferase, bilirubin, hemoglobin, PLR, and SII between the onset of acute pancreatitis and follow-up assessment in patients with CDRAP (*p* < 0.05, Table 4).

**Table 4:** Comparison of biochemical findings between acute pancreatitis onset and follow-up assessment in patients with CDRAP.

Variables	Acute pancreatitis onset	Follow-up	t/Z	*p*-value
Serum AMY, U/L	435.40 ± 415.02	122.00 ± 6.92	−2.78	0.006
Serum LIP, U/L	838.45 ± 683.39	289.50 ± 196.88	−1.49	0.135
Total cholesterol, mmol/L	4.08 ± 1.30	3.26 ± 0.86	−2.45	0.014
Triglyceride, mmol/L	1.36 ± 0.72	1.43 ± 0.43	−0.65	0.515
LDL-C, mmol/L	2.53 ± 1.58	1.87 ± 0.47	−1.83	0.067
HDL-C, mmol/L	1.26 ± 0.95	0.96 ± 0.38	−0.68	0.495
Alanine Aminotransferase, U/L	60.23 ± 78.31	16.00 ± 12.96	−4.52	<0.001
Aspartate Aminotransferase, U/L	54.70 ± 56.50	29.55 ± 31.61	−2.68	0.007
Bilirubin, μmol/L	28.96 ± 40.29	12.58 ± 8.12	−2.68	0.110
Creatinine, μmol/L	90.03 ± 126.99	66.15 ± 38.50	−0.32	0.749
Hemoglobin, g/L	107.94 ± 16.03	87.62 ± 25.35	−3.63	<0.001
NLR	5.71 ± 7.58	4.21 ± 4.06	−0.88	0.380
PLR	236.69 ± 150.49	163.24 ± 103.02	−2.26	0.027
SII	1159.76 ± 902.77	685.45 ± 730.84	−2.35	0.019

AMY, serum amylase; LIP, serum lipase; LDL-C, low-density lipoprotein cholesterol; HDL-C, high-density lipoprotein cholesterol; NLR, neutrophil to lymphocyte ratio; PLR, platelet to lymphocyte ratio; SII, systemic immune inflammation index.

### Drugs used in the CDRAP regimen

The classification and frequency of chemotherapeutic agents used in 62 patients with CDRAP are summarized in Supplementary Table 3. Cyclophosphamide, corticosteroids (hormonal agents), vincristine, and doxorubicin were the most frequently used drugs, with 15, 13, 12, and 9 occurrences, respectively. The drug classifications were assigned according to the criteria established by Badalov et al. (22) for drug-induced pancreatitis.

### Risk factors for chemotherapy-related AEs

In addition, the risk factors for chemotherapy-related AEs were also analyzed. In the univariate logistic regression analysis, a history of alcohol consumption, levels of HDL-C, alanine aminotransferase, aspartate aminotransferase, bilirubin, creatinine, and hemoglobin levels were identified as risk factors for chemotherapy-related AEs (*p* < 0.05, Supplementary Table 4). In the multivariate logistic regression analysis, alanine aminotransferase (*p* = 0.016) and bilirubin (*p* = 0.045) were identified as independent risk factors for chemotherapy-related AEs. However, the results demonstrated that none of the CT-derived pancreatic texture analysis features could be identified as significant risk factors for chemotherapy-related AEs.

## Discussion

Combination chemotherapy is the first-line therapy for many cancers, as it sensitizes cancer cells to drugs, modulates different signaling pathways in cancer cells, and has been shown to combat multidrug resistance (7). However, chemotherapy-related AEs are common in clinical practice, such as bone marrow suppression, gastrointestinal symptoms, and neurotoxicity (23). Drug-induced acute pancreatitis is one such rare but significant adverse effect of chemotherapy, which can be challenging and is difficult to diagnose. This type of pancreatitis accounts for approximately 5% of all cases of acute pancreatitis (24, 25). In the present study, we comprehensively analyzed the conventional CT imaging features and texture analysis characteristics of CDRAP. To the best of our knowledge, this is the largest patient cohort with a systematic description of CDRAP imaging features. In addition, the texture analysis characteristics of patients with CDRAP have not been reported in previous studies. This approach enhanced certainty in diagnosing suspected CDRAP while facilitating early therapeutic intervention and improving prognosis and quality of life outcomes.

Among the conventional imaging features assessed in our study, interstitial edematous pancreatitis presentation, focal or diffuse pancreatic enlargement, and heterogeneous enhancement were the most observed CT features. The most likely pathogenesis is the direct toxic effect of chemotherapy drugs, which causes enzyme activation and autodigestion and manifests as interstitial edema and inflammatory infiltration (26). Similarly, among the imaging characteristics of immune checkpoint inhibitor (ICI)-associated pancreatitis, pancreas enlargement and heterogeneous enhancement were also the most observed features (27). We speculate that CDRAP and ICI-associated pancreatitis could be classified as drug-induced pancreatitis and may exhibit some similar imaging presentations. Acute necrotic collections (necrotizing pancreatitis) and main pancreatic duct dilatation were not observed, and normal pancreas morphology was seen in our CT cohort. This suggests that CDRAP mainly presents as interstitial edematous pancreatitis and/or shows a normal pancreas appearance. This is consistent with previous studies, which suggest that CDRAP is mostly a mild form of pancreatitis (25, 28). However, ICI-associated pancreatitis typically demonstrates more severe pancreatic involvement than CDRAP, often presenting as necrotizing pancreatitis on CT (29). In our cohort, the pancreatic changes in CDRAP were often subtle on imaging, and careful detection of mild pancreatic enlargement and peripancreatic inflammatory changes proved valuable when clinical suspicion was high.

Furthermore, texture analysis features of the pancreas were extracted from the CECT images of patients with CDRAP and those with normal pancreatic performance. By performing LASSO regression analysis, this study identified the most discriminative texture features associated with CDRAP diagnosis, thereby establishing a potential imaging biomarker for its diagnosis. In recent years, CT-based texture analysis techniques have been increasingly used to improve the information obtained from medical images and to enhance diagnostic performance in identifying pancreatic lesions (30–32). In the study by Rocca A (30), a radiomics model was developed to automatically identify mild acute pancreatitis from CT images in patients with acute abdominal pain, particularly when clinical and serological findings were inconclusive. Radiomics and texture analysis are high-throughput techniques used to extract information that helps reflect the microscopic alterations behind macroscopic manifestations (30). For example, the GLCM quantifies textural properties by analyzing the spatial relationships of pixel pairs and extracts features such as contrast, correlation, and energy to characterize tissue coarseness and homogeneity (33). In CDRAP, inflammatory-mediated microstructural disruptions (e.g., intermixed edema regions and normal parenchyma) may manifest as alterations in GLCM-derived parameters. In addition, the GLSZM evaluates the size distribution of homogeneous gray-level zones, thereby capturing spatial aggregation of microlesions (e.g., necrotic foci) or edematous regions (33). In CDRAP, localized pathological changes may exhibit an increased proportion of small-sized zones. Given that 32.26% of clinically diagnosed CDRAP patients demonstrated normal pancreatic morphology on CT imaging and that 67.74% exhibited radiological manifestations of acute pancreatitis, a comparative analysis of texture features was conducted between these subgroups. The analysis revealed that three GLSZM features, one first-order statistical feature, and one GLCM feature differed between the patients with CT findings suggestive of acute pancreatitis and those with a normal pancreatic appearance. This suggests that in a small proportion of clinically diagnosed CDRAP patients with normal pancreatic morphology on CT imaging, the disease may be detectable through changes in these texture features.

In our cohort, approximately 87.10% of the patients reported pain in the upper abdomen, suggesting that this symptom is a common clinical presentation of CDRAP. The time from the initiation of chemotherapy to the onset of clinical symptoms was 181.33 days, and there were approximately three to four chemotherapy cycles. The time from the onset of clinical symptoms to imaging was 66.33 h, reflecting an inevitable delay. Therefore, timely CECT scans are particularly recommended for patients undergoing chemotherapy who present with pain symptoms and biochemical evidence of pancreatic injury, especially after three to four chemotherapy cycles. The American College of Radiology Appropriateness Criteria suggests that CECT provides a comprehensive assessment for the diagnosis, severity evaluation, and identification of potential complications when acute pancreatitis is suspected (29, 34, 35).

Furthermore, in the present study, 30 out of 62 (48.39%) patients with CDRAP experienced chemotherapy-related AEs, and 18 out of 34 (52.94%) patients without CDRAP experienced chemotherapy-related AEs. These AEs included hematologic toxicity (anemia, thrombocytopenia), gastrointestinal toxicity (hepatic dysfunction, stomatitis, mucositis), renal toxicity (renal impairment), and neurotoxicity (peripheral neuropathy or central neurotoxicity). The present study evaluated potential risk factors for chemotherapy-related AEs among biochemical parameters and radiomic texture analysis features and found that elevated levels of alanine aminotransferase (ALT, *p* = 0.016) and bilirubin (*p* = 0.045) were independent risk factors for chemotherapy-related AEs. This highlights the importance of pre-chemotherapy laboratory tests.

In the follow-up imaging examination, 21 (33.87%) patients developed pancreatic atrophy, and 19.05% (4/21) of these patients experienced pancreatic exocrine dysfunction compared to the baseline examination. Interestingly, among the 10 patients with normal pancreas on initial imaging, 4 developed acute pancreatitis changes on follow-up CT scans. This indicates that baseline imaging might reveal only mild and subtle pancreatic changes that become more apparent as the disease progresses.

In the study by Badalov et al. (22), drugs that have been reported to cause acute pancreatitis were classified based on the published weight of evidence for each agent and the pattern of clinical presentation. In our study, all commonly used chemotherapy drugs in patients with CDRAP were listed in the drug classification categories. For instance, dexamethasone (number of patients receiving the drug, *n* = 5; Class Ib), pegaspargase (*n* = 3; Class II), prednisone (*n* = 13; Class III), cyclophosphamide (*n* = 15; Class III), and doxorubicin (*n* = 9; Class III), among others were included. This study demonstrated concordance with prior evidence on the risk of drug-associated pancreatitis. Together, these results summarize five major types of mechanisms contributing to CDRAP—structural, toxin, metabolic, vascular, and others (22). Uncoincidentally, the administration of a combination of drugs can lead to more severe ICI-associated AEs, as reported in previous research (27).

There are several limitations to our study. First, we were unable to acquire baseline normal pancreatic imaging to perform a rechallenge test in order to clarify the causal relationship between the use of drugs and the onset of acute pancreatitis. In addition, we did not adhere to a predetermined schedule for follow-up imaging. According to some imaging findings, follow-up scans may reveal missed changes in the pancreas. Second, the single-center retrospective design with a limited cohort size lacks external validation, and inter-scanner/texture analysis standardization requires further investigation, as variations in acquisition parameters may affect the stability of feature extraction. However, to the best of our knowledge, this is the largest patient cohort with a systematic description of CDRAP imaging features. Third, some patients had other risk factors for pancreatitis; however, in each case, there was no clinical, biochemical, and/or imaging evidence of pancreatitis before chemotherapy. Whether these risk factors contribute to CDRAP requires further prospective studies. Finally, due to the lack of long-term follow-up data, we were unable to evaluate long-term imaging, pancreatic function, and prognosis However, given the heterogeneity of primary cancer types and disease stages among patients with CDRAP, it is unlikely that CDRAP significantly affects long-term prognosis.

## Conclusion

CDRAP mainly presented as interstitial edematous pancreatitis with or without normal pancreatic morphology. Imaging texture analysis may serve as a potential biomarker for its detection. By combining conventional imaging features with texture analysis characteristics, this approach has the potential to assist radiologists and clinicians in the identification of CDRAP. Future multi-center studies with large sample sizes are needed to validate the generalizability of findings, establish a standardized texture analysis process, and explore the feasibility of clinical translation.

## Data Availability

The raw data supporting the conclusions of this article will be made available by the authors, without undue reservation.

## Footnotes

^1^ https://www.slicer.org
