# Supplementary material for: Computed tomography-based conventional imaging features and texture analysis characteristics of chemotherapy drug-related acute pancreatitis
Source: Front Med (Lausanne). 2025 May 12;12:1497944. doi: 10.3389/fmed.2025.1497944 (PMC12104288; doi:10.3389/fmed.2025.1497944)
Supplement: Supplementary file 1 [file Data_Sheet_1.docx]

**Supplementary Table 1** Texture features with a significant difference between patients with and without CDRAP and categories.

| Variables | Categories |
| --- | --- |
| auto__log-sigma-1-0-mm-3D_glszm_GrayLevelNonUniformity | GLSZM |
| auto__log-sigma-3-0-mm-3D_glszm_HighGrayLevelZoneEmphasis | GLSZM |
| auto__logarithm_glszm_SizeZoneNonUniformity | GLSZM |
| auto__square_firstorder_10Percentile | First order |
| auto__wavelet-HHH_glcm_SumEntropy | GLCM |
| auto__wavelet-HHL_glcm_SumEntropy | GLCM |
| auto__wavelet-HLH_glcm_Imc2 | GLCM |
| auto__wavelet-HLL_glrlm_LongRunEmphasis | GLRLM |
| auto__wavelet-LHH_glcm_DifferenceEntropy | GLCM |
| auto__wavelet-LHL_glszm_SizeZoneNonUniformity | GLSZM |
| auto__wavelet-LHL_glszm_SizeZoneNonUniformityNormalized | GLSZM |
| auto__wavelet-LHL_glszm_SmallAreaLowGrayLevelEmphasis | GLSZM |
| auto__wavelet-LLH_glcm_Correlation | GLCM |
| auto__wavelet-LLH_gldm_DependenceEntropy | GLDM |
| auto__wavelet-LLH_glrlm_HighGrayLevelRunEmphasis | GLRLM |
| auto__wavelet-LLH_glrlm_LongRunEmphasis | GLRLM |
| auto__wavelet-LLL_glszm_SizeZoneNonUniformity | GLSZM |

Note: GLSZM, Gray Level Size Zone Matrix; GLCM, Gray Level Co-occurrence Matrix; GLRLM, Gray Level Run Length Matrix; GLDM, Gray Level Dependence Matrix.

**Supplementary Table 2** Comparison of texture analysis features between patients with CT findings suggestive of acute pancreatitis and those with normal pancreatic appearance in patients with CDRAP

| Variables | Acute pancreatitis changes in patients with CDRAP (n=42) | Normal appearance of pancreas in patients with CDRAP  (n=20) | t/Z/χ^2^ | *P* |
| --- | --- | --- | --- | --- |
| auto__log-sigma-1-0-mm-3D_glszm_GrayLevelNonUniformity | 21.82±9.63 | 18.57±10.33 | -1.42 | 0.157 |
| auto__log-sigma-3-0-mm-3D_glszm_HighGrayLevelZoneEmphasis | 3.16±0.63 | 3.26±0.43 | -0.21 | 0.831 |
| auto__logarithm_glszm_SizeZoneNonUniformity | 11.25±9.40 | 5.33±6.79 | -3.05 | 0.002 |
| auto__square_firstorder_10Percentile | 0.05±0.08 | 0.07±0.07 | -2.53 | 0.011 |
| auto__wavelet-HHH_glcm_SumEntropy | 1.44±0.01 | 1.44±0.02 | -0.23 | 0.819 |
| auto__wavelet-HHL_glcm_SumEntropy | 1.43±0.01 | 1.44±0.02 | -1.66 | 0.101 |
| auto__wavelet-HLH_glcm_Imc2 | 0.25±0.04 | 0.25±0.06 | -0.02 | 0.983 |
| auto__wavelet-HLL_glrlm_LongRunEmphasis | 5.98±0.70 | 5.87±0.83 | 0.56 | 0.580 |
| auto__wavelet-LHH_glcm_DifferenceEntropy | 0.94±0.01 | 0.93±0.01 | 0.67 | 0.503 |
| auto__wavelet-LHL_glszm_SizeZoneNonUniformity | 2.78±1.13 | 2.22±0.90 | -2.62 | 0.008 |
| auto__wavelet-LHL_glszm_SizeZoneNonUniformityNormalized | 0.15±0.04 | 0.14±0.05 | 0.35 | 0.728 |
| auto__wavelet-LHL_glszm_SmallAreaLowGrayLevelEmphasis | 0.18±0.06 | 0.17±0.10 | 0.64 | 0.525 |
| auto__wavelet-LLH_glcm_Correlation | 0.48±0.10 | 0.49±0.16 | -0.48 | 0.630 |
| auto__wavelet-LLH_gldm_DependenceEntropy | 3.52±0.51 | 3.51±0.34 | -1.02 | 0.306 |
| auto__wavelet-LLH_glrlm_HighGrayLevelRunEmphasis | 2.42±0.13 | 2.44±0.12 | -0.66 | 0.512 |
| auto__wavelet-LLH_glrlm_LongRunEmphasis | 29.57±17.32 | 29.82±27.64 | -1.15 | 0.252 |
| auto__wavelet-LLL_glszm_SizeZoneNonUniformity | 2.33±1.56 | 1.63±1.08 | -2.04 | 0.042 |

Note: CDRAP, chemotherapy drug-related acute pancreatitis.

**Supplementary Table 3** Combination chemotherapy regimen drug of CDRAP

| Drugs | Number of patients received the drug | Drug classification according to Badalov et al. (2007) |
| --- | --- | --- |
| Alkylating Agents |  |  |
| Cyclophosphamide | 15 | Class III |
| S-1 | 3 | NA |
| Tegafur | 1 | NA |
| Platinum Drugs |  |  |
| Cisplatin | 4 | Class III |
| Oxaserum lipaselatin | 4 | NA |
| Nedaplatin | 2 | NA |
| Anthracyclines |  |  |
| Docetaxel | 5 | NA |
| Serum lipaseosomal Paclitaxel | 4 | Class III |
| Anthracyclines/Anthraquinones |  |  |
| Doxorubicin | 9 | Class III |
| Epirubicin | 3 | NA |
| Serum lipaseosomal Doxorubicin | 1 | NA |
| Antimetabolites |  |  |
| Fluorouracil (5-FU) | 1 | NA |
| Gemcitabine | 1 | NA |
| Methotrexate | 2 | Class III |
| Calcium Folinate (Leucovorin) | 1 | NA |
| Plant Alkaloids |  |  |
| Vincristine | 12 | NA |
| Vindesine | 1 | NA |
| Topoisomerase Inhibitors |  |  |
| Etoposide | 3 | Class III |
| Corticosteroids (Hormonal Agents) |  |  |
| Prednisone | 13 | Class III |
| Dexamethasone | 5 | Class Ib |
| Enzyme/Biological Agents |  |  |
| Pegaspargase | 3 | Class II |

**Supplementary Table 4** Logistic regression analysis of chemotherapy-related AEs in patients underwent chemotherapy

|  | Univariate analysis | | |  | Multivariate analysis | | |
| --- | --- | --- | --- | --- | --- | --- | --- |
| Variables | OR | 95%CI | *P* |  | OR | 95%CI | *P* |
| Age, years | 0.997 | 0.975,1.019 | 0.770 |  |  |  |  |
| Hypertension, n (%) | 0.810 | 0.328,1.996 | 0.646 |  |  |  |  |
| History of alcohol consumption, n (%) | 3.818 | 1.081,13.486 | 0.037 |  | 0.494 | 0.009,26.838 | 0.730 |
| History of smoking, n (%) | 1.486 | 0.590,3.744 | 0.401 |  |  |  |  |
| History of cholelithiasis, n (%) | 0.500 | 0.085,2.954 | 0.444 |  |  |  |  |
| Hypertriglyceridemia, n (%) | 0.800 | 0.266,2.407 | 0.691 |  |  |  |  |
| Primary cancer, n (%) | 0.869 | 0.727,1.037 | 0.119 |  |  |  |  |
| Number of chemotherapy cycle | 0.167 | 0.750,1.051 | 0.888 |  |  |  |  |
| Total cholesterol, mmol/L | 1.017 | 0.711,1.454 | 0.928 |  |  |  |  |
| Triglyceride, mmol/L | 1.763 | 0.862,3.606 | 0.121 |  |  |  |  |
| LDL-C, mmol/L | 0.973 | 0.703,1.348 | 0.871 |  |  |  |  |
| HDL-C, mmol/L | 0.256 | 0.085,0.769 | 0.015 |  | 0.099 | 0.009,1.148 | 0.064 |
| Alanine Aminotransferase, U/L | 1.019 | 1.003,1.035 | 0.020 |  | 1.119 | 1.021,1.227 | 0.016 |
| Aspartate Aminotransferase, U/L | 1.012 | 1.001,1.023 | 0.037 |  | 0.998 | 0.941,1.060 | 0.957 |
| Bilirubin, μmol/L | 1.018 | 1.002,1.035 | 0.026 |  | 0.904 | 0.819,0.998 | 0.045 |
| Creatinine, μmol/L | 1.005 | 0.997,1.013 | 0.211 |  |  |  |  |
| Hemoglobin, g/L | 0.978 | 0.956,1.000 | 0.054 |  |  |  |  |
| NLR | 1.046 | 0.979,1.117 | 0.183 |  |  |  |  |
| PLR | 1.000 | 0.998,1.001 | 0.624 |  |  |  |  |
| SII | 1.000 | 1.000,1.000 | 0.972 |  |  |  |  |
| auto__log-sigma-1-0-mm-3D_glszm_GrayLevelNonUniformity | 1.019 | 0.975,1.065 | 0.406 |  |  |  |  |
| auto__log-sigma-3-0-mm-3D_glszm_HighGrayLevelZoneEmphasis | 1.003 | 0.550,1.829 | 0.992 |  |  |  |  |
| auto__logarithm_glszm_SizeZoneNonUniformity | 1.044 | 0.992,1.099 | 0.101 |  |  |  |  |
| auto__square_firstorder_10Percentile | 0.952 | 0.012,74.311 | 0.982 |  |  |  |  |
| auto__wavelet-HHH_glcm_SumEntropy |  |  | NS |  |  |  |  |
| auto__wavelet-HHL_glcm_SumEntropy |  |  | NS |  |  |  |  |
| auto__wavelet-HLH_glcm_Imc2 |  |  | NS |  |  |  |  |
| auto__wavelet-HLL_glrlm_LongRunEmphasis | 1.08 | 0.650,1.795 | 0.765 |  |  |  |  |
| auto__wavelet-LHH_glcm_DifferenceEntropy | 1.355 | 0.740,2.481 | 0.324 |  |  |  |  |
| auto__wavelet-LHL_glszm_SizeZoneNonUniformity | 1.041 | 0.793,1.366 | 0.774 |  |  |  |  |
| auto__wavelet-LHL_glszm_SizeZoneNonUniformityNormalized |  |  | NS |  |  |  |  |
| auto__wavelet-LHL_glszm_SmallAreaLowGrayLevelEmphasis | 2.628 | 0.365,18.939 | 0.388 |  |  |  |  |
| auto__wavelet-LLH_glcm_Correlation | 1.049 | 0.778,1.416 | 0.752 |  |  |  |  |
| auto__wavelet-LLH_gldm_DependenceEntropy | 1.165 | 0.651,2.084 | 0.608 |  |  |  |  |
| auto__wavelet-LLH_glrlm_HighGrayLevelRunEmphasis | 1.006 | 0.993,1.019 | 0.357 |  |  |  |  |
| auto__wavelet-LLH_glrlm_LongRunEmphasis | 0.992 | 0.973,1.011 | 0.382 |  |  |  |  |
| auto__wavelet-LLL_glszm_SizeZoneNonUniformity | 1.045 | 0.742,1.473 | 0.799 |  |  |  |  |

Note: CDRAP, chemotherapy drug-related acute pancreatitis; AMY, serum amylase; LIP, serum lipase; LDL-C, low-density serum lipaseoprotein-cholesterol; HDL-C, high-density serum lipaseoprotein-cholesterol; NLR, neutrophil to lymphocyte ratio; PLR, platelet to lymphocyte ratio; SII, systemic immune inflammation index; AE, adverse event.


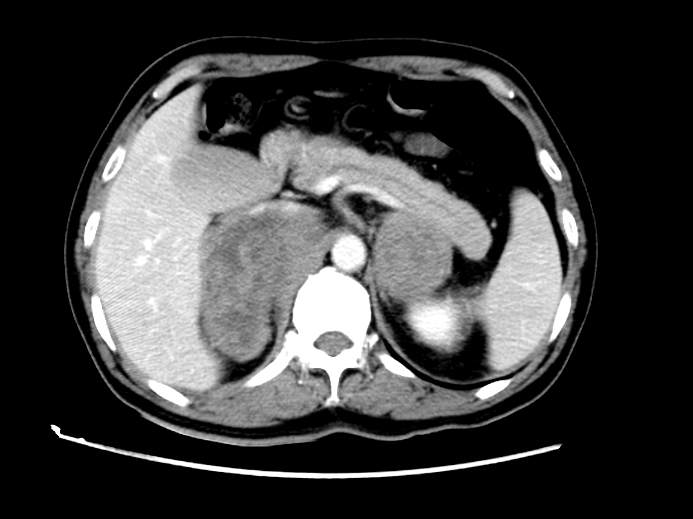

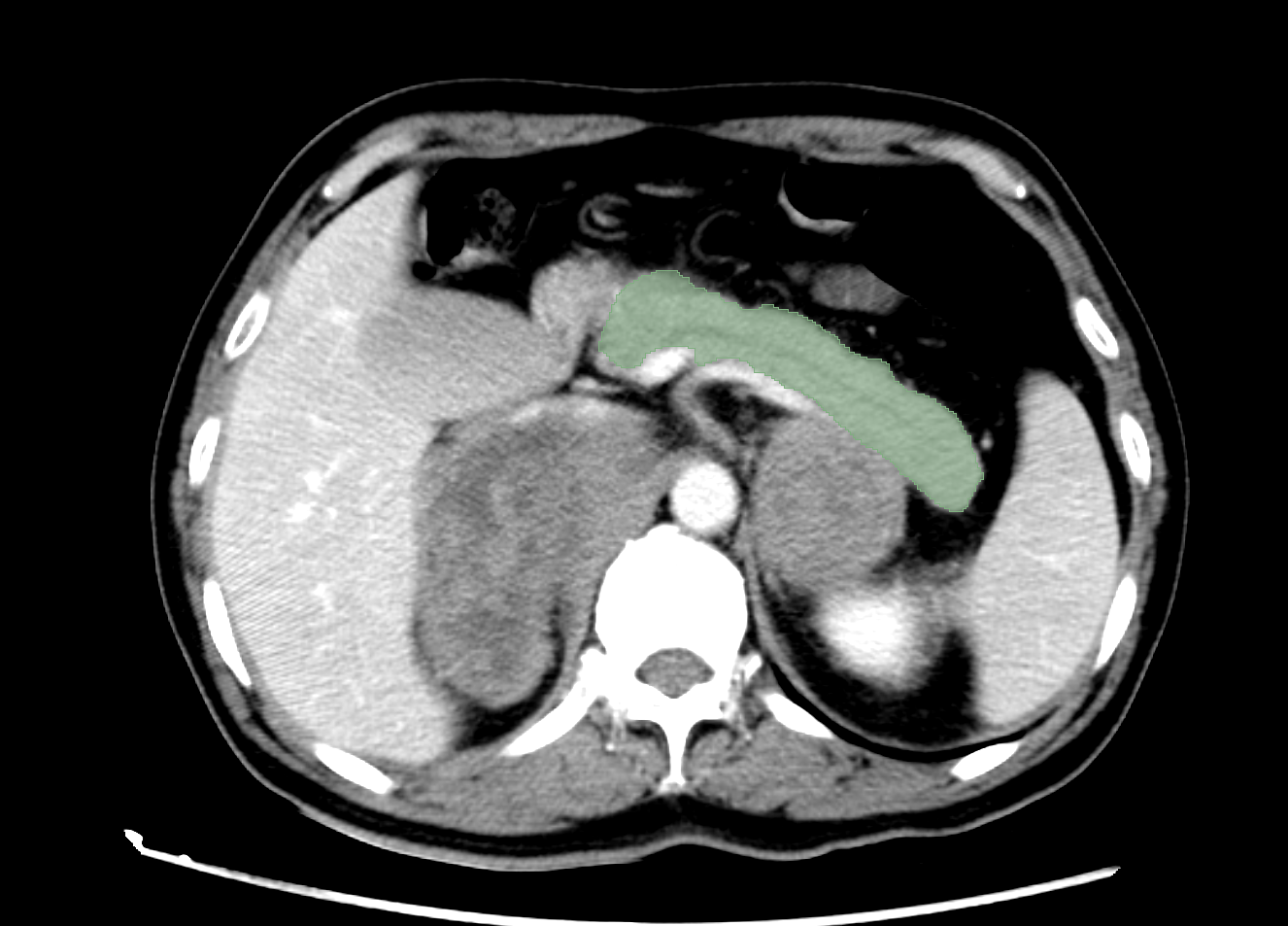


**Supplementary Fig.1.** Example of pancreatic ROI outlining on a CT slice in portal phase.


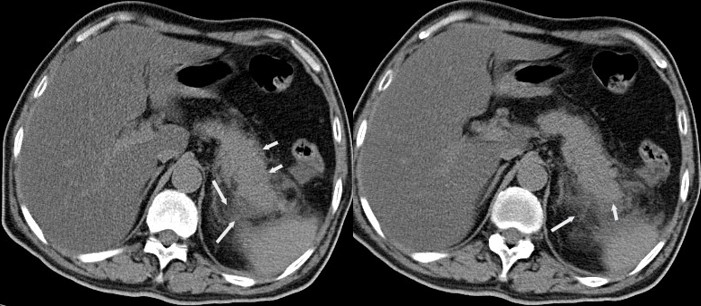


**Supplementary Fig.2.** A 58-year-old man with peripheral T-cell non-Hodgkin lymphoma. He received CHOP regimen for 3 cycles with AMY=170 U/L and LIP=695 U/L. CT presented focal pancreas enlargement (short arrows) and peripancreatic stranding (long arrows). CT also showed a diffuse decrease in liver density compared with spin density, which eventually identified drug-induced hepatitis.


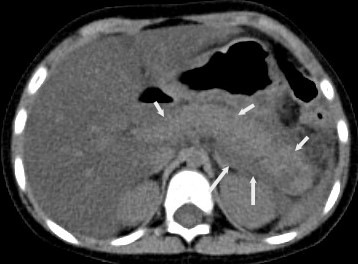


**Supplementary Fig.3.** A 4-year-old girl with ALL. She received the VDLP regimen for 2 cycles with AMY=888 U/L and LIP=300 U/L. CT presented diffuse pancreas enlargement (short arrows) and peripancreatic fluid collection (long arrows).


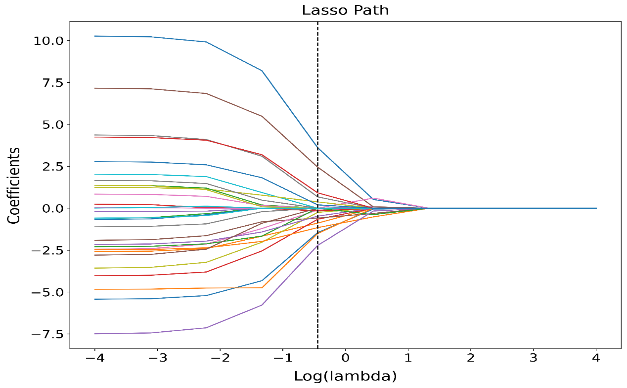

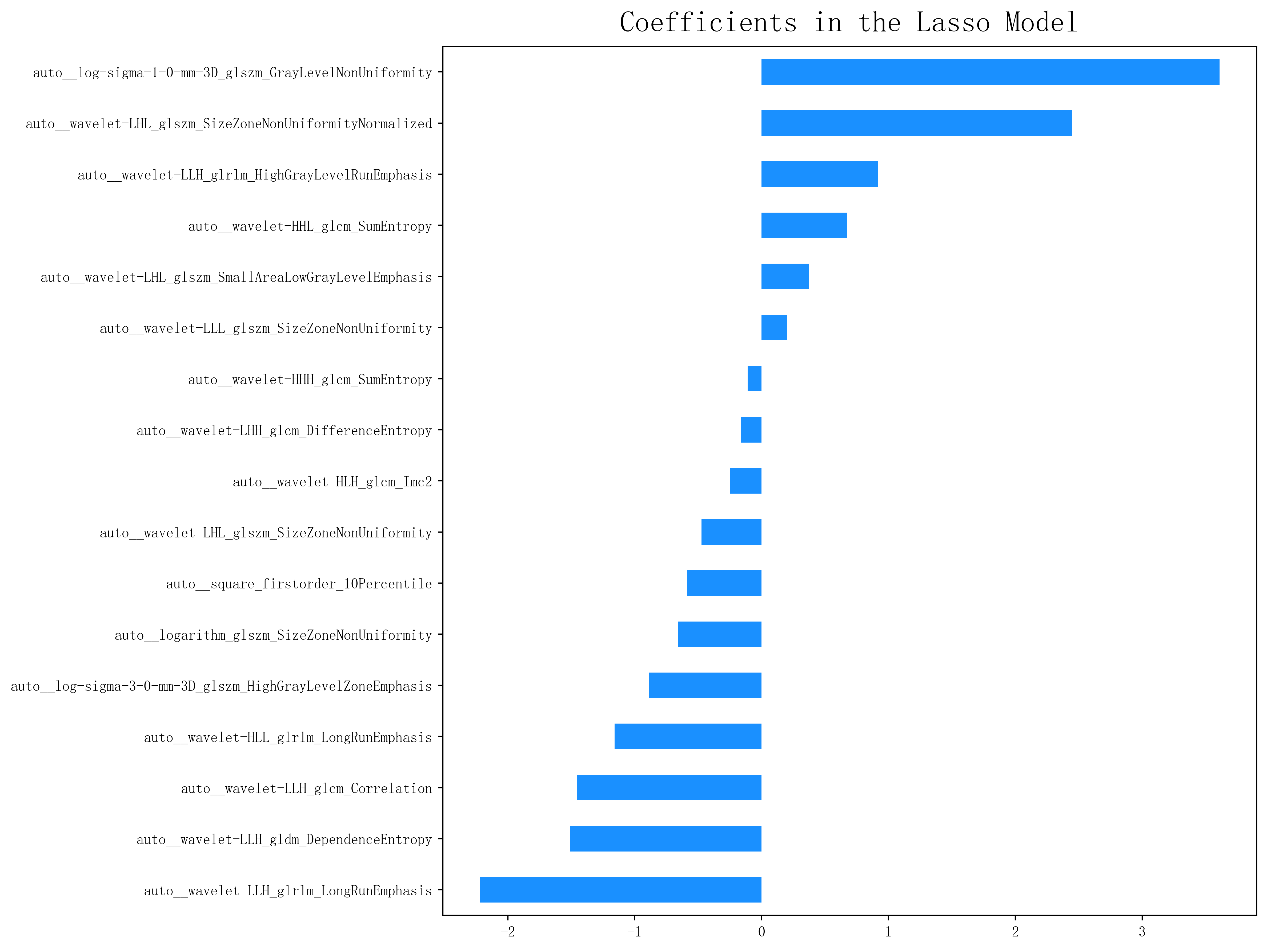


**Supplementary Fig.4.** Lasso regression and texture features with a significant difference between patients with and without CDRAP.

**
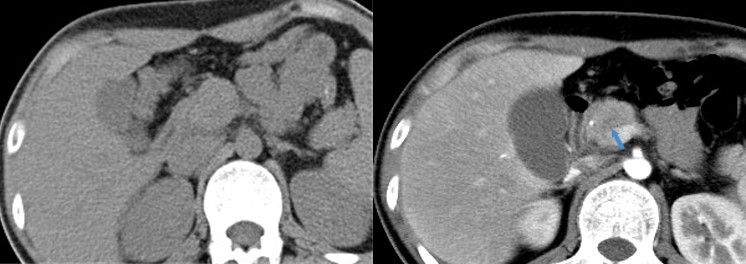
**

**Supplementary** **Fig.5.** A 19-year-old man with NK/T cell lymphoma. He received the gemcitabine, olisaplatin, and pegaspargase regimens of 2 cycles with AMY=130 U/L and LIP=1877 U/L. CT (2017-8-22, right) presented a normal pancreas appearance. Then, the patient stopped chemotherapy and received symptomatic treatment for pancreatitis, and CT (2017-9-13, left, arrow) showed heterogeneous enhancement.
